# Validity of predictive factors of acute complicated appendicitis

**DOI:** 10.1186/s13017-016-0107-0

**Published:** 2016-09-26

**Authors:** Yuki Imaoka, Toshiyuki Itamoto, Yuji Takakura, Takahisa Suzuki, Satoshi Ikeda, Takashi Urushihara

**Affiliations:** 1 Department of Gastroenterological Surgery, Hiroshima Prefectural Hospital, 5-54, Ujinakanda, Minami-ku, Hiroshima 734-00041 Japan; 2 Department of Gastroenterological and Transplant Surgery, Applied Life Sciences, Institute of Biomedical & Health Sciences, Hiroshima University, 1-2-3, Kasumi, Minami-ku, Hiroshima 734-8551 Japan

**Keywords:** Acute appendicitis, Predictive factor, Emergency surgery

## Abstract

**Background:**

Our previous retrospective study revealed the three preoperative predictors of complicated appendicitis (perforated or gangrenous appendicitis), which are body temperature ≥37.4 °C, C-reactive protein ≥4.7 mg/dl, and fluid collection surrounding the appendix on computed tomography. We reported here an additional prospective study to verify our ability to predict complicated appendicitis using the three preoperative predictors and thus facilitate better informed decisions regarding emergency surgery during night or holiday shifts.

**Methods:**

We prospectively evaluated 116 adult patients who underwent surgery for acute appendicitis from January 2013 to October 2014. Ninety patients with one or more predictive factors of complicated appendicitis underwent immediate surgery regardless of the time of patient’s presentation. Twenty-six patients had no predictive factors and thus were suspected to have uncomplicated appendicitis. Of the 26 patients, 14 who presented to our hospital during office hours underwent immediate surgery. The other 12 patients who presented to our hospital at night or on a holiday underwent short, in-hospital delayed surgery during office hours.

**Results:**

All patients with no predictive factors had uncomplicated appendicitis, whereas 37 %, 81 %, and 100 % of patients with one, two, or all three factors, respectively, were diagnosed with complicated appendicitis. The emergency operation rate decreased from 83 % before to 58 % after adopting this scoring system, but no significant differences in postoperative complication rates and hospitalization periods were observed.

**Conclusions:**

The above-mentioned preoperative factors predictive of complicated appendicitis preoperatively are useful for emergency surgical decisions and reduce the burdens on surgeons and medical staff.

## Background

Acute appendicitis is the most well-known acute abdominal disease. However, not all diagnosed cases of acute appendicitis require emergency surgery. Non-operative management is recommended for uncomplicated appendicitis [1], but preoperative distinction between uncomplicated and complicated disease is challenging. In addition, cases of complicated appendicitis, which include perforated appendicitis and gangrenous appendicitis, may progress to acute peritonitis, a condition that necessitates emergency surgery regardless of the time of development. This emergent nature presents additional complications, as our hospital is staffed by young surgical residents (3–5 years after graduation) at night and over holidays, who examine patients and make decisions regarding the indications for emergency surgeries (e.g., appendectomy). In contrast, the short-term risk of perforation in cases of uncomplicated appendicitis, such as catarrhal and cellulitis appendicitis is low, and these cases can be treated conservatively with antibiotics until sufficient on-duty medical staffs are available to perform surgery. In addition, some of these cases can continue receiving conservative treatment with antibiotics [2–4].

To address the challenge presented by the emergent nature of some appendicitis cases, we performed a retrospective study in which we considered three factors, a body temperature ≥37.4 °C, C-reactive protein (CRP) level ≥4.7 mg/dl, and fluid collection surrounding the appendix on computed tomography (CT), as potential preoperative factors predictive of complicated appendicitis [5]. Herein, we report an additional prospective study to verify our ability to predict complicated appendicitis using these factors and thus facilitate better informed decisions regarding emergency surgery during night or holiday shifts.

## Methods

**Fig. 1:**
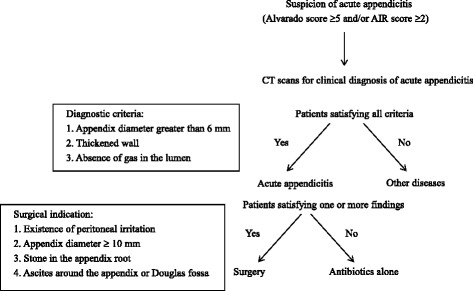
Algorithm indicating the diagnosis and treatment strategies for acute appendicitis

Our strategies of the diagnostic strategies of and for acute appendicitis are shown in Fig. 1. Clinical suspicion of acute appendicitis is made based on the routine use of Alvarado [6] and appendicitis inflammatory response (AIR) scores [7]. In the absence of contraindication to CT use such as pregnancy, CT scans are performed for patients with an Alvarado score ≥ of 5 or more and/or AIR score ≥ of 2 or more, if patients had no contraindication of use of CT scan such as pregnancy. A diagnosis of acute appendicitis is given if the patient has when positive CT findings on all of the following CT findings: a short appendix diameter greater than >6 mm, a thickened wall of the appendix, and absence of gas in the appendicular lumen. Decisions to surgery was performed when the patient was positive for at least one of the following findings: the existence of peritoneal irritation, a short appendix diameter ≥10 mm, stone in the appendix root, and ascites around the appendix or Douglas fossa. Patients without these factors received non-operative treatment.

**Fig. 2:**
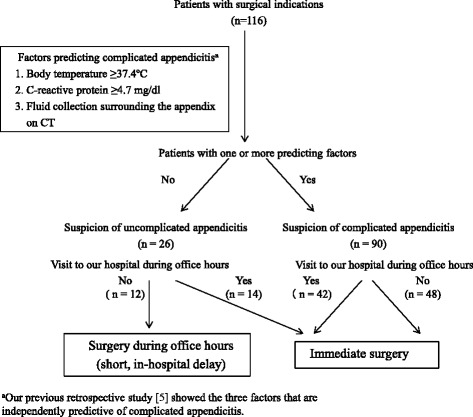
Algorithm indicating the timing of surgery according to the predictive factors of uncomplicated appendicitis

We prospectively evaluated 116 patients who underwent surgery for acute appendicitis from January 2013 to October 2014 in this study. Patients who were treated successfully with antibiotics were excluded. Out of the 116 patients, 90 patients who had one or more factors predictive of complicated appendicitis underwent the immediate surgery regardless of the time of the patients’ visited to our hospital. Twenty-six patients had no predictive factors and thus, whose appendicitis were suspected to have be uncomplicated appendicitis. Out of the 26 patients, 14 patients who presented to our hospital during office hours underwent the immediate surgery. The other 12 patients who presented to our hospital at night or on a holiday underwent delayed surgery during office hours (Fig. 2).

Histopathologically, catarrhal appendicitis was defined as the apparent enlargement of lymphoid follicles in the appendix mucosa, and cellulitis appendicitis was defined as neutrophil infiltration into all layers. Gangrenous appendicitis was defined as neutrophil infiltration and muscle layer necrosis, and perforated appendicitis was defined as necrosis and perforation in all layers. Complicated appendicitis was defined as a pathologically proven gangrenous or perforated appendix. Our strategies for patients with acute appendicitis indicated for surgery included immediate operation for patients with suspicion of complicated appendicitis and short, in-hospital delay for patients with suspicion of uncomplicated appendicitis.

JMP statistical software (JMP® 11; SAS Institute Inc., Cary, NC, USA) was used for the statistical analysis. A *p*-value ≤0.05 was considered statistically significant. Pearson’s chi-square test was used to determine the significance of differences between dichotomous groups. Fisher’s exact test was used when a table included a cell with an expected frequency of <5.

## Results

The prospective study included 65 male (56 %) and 51 female patients (44 %). The general patient characteristics are shown in Table 1. The mean patient age was 44.5 years, with a range of 14–90 years. Overall, 52 (45 %) of the 116 patients had uncomplicated appendicitis: 2 had pathologically proven catarrhal appendicitis and 50 had pathologically proven cellulitis appendicitis. The remaining 64 patients (55 %) had complicated appendicitis. All patients without any of the three predictive factors (body temperature ≥37.4 °C, CRP level ≥4.7 mg/dl, and fluid surrounding the appendix on CT) had uncomplicated appendicitis. In contrast, 37 %, 81 %, and 100 % of the patients with one, two, or all three factors, respectively, were proved pathologically to have complicated appendicitis (Table 2).

**Table 1:** Patient characteristics

Mean age (ranges), years	44.5 (14–90)
Male/female	65/51
During office hour/at night or on a holiday^a^	56/60
Body temperature (°C)	37.4 (35.8–40)
WBC (/μl)	12000 (2700–25700)
CRP (mg/dl)	5.15 (0.2–36.0)
Fluid collection surrounding appendix +/-	66/50
Uncomplicated/complicated	52/64
Operation	
Laparotomy	71
Laparoscopy	42
Ileocecal resection	3

^a^The time when the patients presented to our hospital

**Table 2:** Relationship between the number of predictive factor and the severity of appendicitis

Number of predictive factor	0	1	2	3
Uncomplicated (*n* = 52)	26 (100 %)	19 (63 %)	7 (19 %)	0 (0 %)
Complicated (*n* = 64)	0 (0 %)	11 (37 %)	29 (81 %)	24 (100 %)
Total (*n* = 116)	26	30	36	24

During the prospective study conducted after adopting this scoring system, 35 (58 %) of the 60 patients admitted to the hospital at night or over a holiday underwent immediate surgery. This represented a decrease of 25 percentage points from the immediate surgery rate of 83 % during the retrospective study period of January 2009 to December 2012 (172 cases). However, there were no significant differences in the postoperative complication rate and hospitalization period between the prospective and retrospective studies (Tables 3 and 4).

**Table 3:** Pathological findings and postoperative outcomes

	January 2009 to December 2012 (172 cases, retrospective study) [5]	January 2013 to October 2014 (116 cases, prospective study)	*p*-Value
Severity of appendicitis (uncomplicated/complicated)	120/52	52/64	<0.01
Hospital stay	5 (3–31)	4 (3–22)	N.S.
Postoperative complications	26 (15 %)	21 (18 %)	N.S.

Pathological findings of the resected appendix and postoperative outcomes compared with those of previously published retrospective data

N.S., not significant

**Table 4:** Immediate operation rates at night or on holiday

	January 2009 to December 2012 (172 cases, retrospective study) [5]	January 2013 to October 2014 (116 cases, prospective study)	*p*-Value
During office hour/at night or on a holiday^a^	113/59	56/60	< 0.01
Immediate operation rates at night or on holiday	49 (83 %)	35 (58 %)	< 0.01

Results of intentional prevention from immediate surgery at night or on a holiday compared with those of retrospective study when without the intention

^a^The time when the patients presented to our hospital

## Discussion

The Alvarado and AIR scores are standardized diagnostic approaches in evaluating patients with suspected acute appendicitis, using only clinical signs and symptoms and laboratory values. Di Saverio et al. suggested that the combination of scores might significantly reduce the risk of overpredicting acute appendicitis and reach a diagnostic performance as highly reliable as a CT scan, thus avoiding the routine use of CT [8]. Moreover, they emphasized that both scores were the only independent predictive factors of non-operative management failure with antibiotics for uncomplicated appendicitis [8].

The treatment of patients with complicated intra-abdominal infection involves both timely source control and antimicrobial therapy [9]. Clinical trials have demonstrated the successful treatment of acute appendicitis with antibiotics [4, 10–12]. Notably, not all cases of appendicitis can be treated surgically, especially cases involving catarrhal appendicitis [13], and unnecessary surgeries should be avoided in light of the risk complications such as ileus (1.2 % of cases) and abdominal hernia (0.68 % of cases) [14]. However, cases of complicated appendicitis, such as perforated appendicitis and gangrenous appendicitis, can potentially progress to acute peritonitis, which necessitates emergency surgery. Cases of complicated appendicitis with localized abscesses, however, present a lower risk of progression to acute peritonitis, allowing surgery to be delayed until normal office hours, and recent studies of this protocol, or interval appendectomy, have confirmed the safety of this approach [3, 15].

The surgical indication criteria for acute appendicitis in our department are shown in Fig. 1. Some of the patients with uncomplicated appendicitis and all of the patients with complicated appendicitis had surgical indication according to our criteria. Although cases of complicated appendicitis should be treated immediately, it remains a question whether cases of uncomplicated appendicitis indicated for surgical treatment should be treated immediately even at night or on a holiday.

Although several previous reports have discussed factors associated with the diagnosis of acute appendicitis, the ability of preoperative factors in predicting the presence of complicated appendicitis is not easy to verify [6, 16–18]. However, Atema et al. [19] reported that the scoring system accurately predicted the complicated appendicitis using a maximum possible score of 22 points based on clinical and CT features and a model was created that included age, body temperature, duration of symptoms, white blood cell count, C-reactive protein level, and presence of extraluminal free air, periappendiceal fluid, and appendicolith. Of the 284 patients, 150 had a score of 6 points or less, of whom eight (5.3 %) had complicated appendicitis, giving a negative predictive value (NPV) of 94.7 %. Herein, we report another simple scoring system predicting the complicated appendicitis.

To better identify preoperative predictive factors of complicated appendicitis, we conducted a retrospective and a prospective study to determine the validity of three potential factors (body temperature ≥37.4 °C, CRP ≥4.7 mg/dl, and fluid collection surrounding the appendix on CT) [5]. We performed a receiver operating characteristic (ROC) analysis to identify the most sensitive cut-off level and used multivariate logistic regression analysis to investigate these three predictive values for clinical events in the retrospective study [5]. In the prospective study, we were able to exclude all cases of uncomplicated appendicitis using these predictive factors. Similarly, we could exclude all cases of complicated appendicitis by selecting cases with no predictive factors, giving an NPV of 100 %. In these latter cases, indicated procedures could be postponed to avoid surgeries at night or over holidays. Moreover, a short, in-hospital delay for uncomplicated appendicitis indicated for surgery has proved to be a safe procedure. However, the discrimination of cases with only one or two predictive factors remains controversial, and further prospective study is needed to support decisions regarding emergency surgery in such cases.

After adopting our scoring system, we observed an increase in the frequency of complicated appendicitis, and we expected that the number of patients treated successfully with antibiotics also increased. Non-operative management would be an alternative for uncomplicated appendicitis if cases of complicated appendicitis can be excluded prior to surgery. However, we also recognized some bias in this study, as we excluded patients who were treated successfully with antibiotics from the trial, because we have no way to know their actual pathology. We observed a statistically significant reduction in the frequency of immediate surgery among cases admitted at night or on holidays from 83 % to 58 % after this scoring system was adopted, indicating an effective reduction in the burden placed on surgeons and medical staff. Recently, the strategy of short, in-hospital delay for uncomplicated appendicitis indicated for surgery has been recommended in the World Society of Emergency Surgery Jerusalem guidelines for diagnosis and treatment of acute appendicitis [1].

## Conclusions

In conclusion, the three factors, body temperature ≥37.4 °C, C-reactive protein ≥4.7 mg/dl, and fluid collection surrounding the appendix on CT, are useful in predicting cases of complicated appendicitis preoperatively and can thus facilitate decisions regarding emergency surgery. The scoring system can avoid emergency surgery at night or on a holiday and lead to non-operative management.

## Abbreviations

AIR: Appendicitis inflammatory response
CRP: C-reactive protein
CT: Computed tomography
NPV: Negative predictive value
ROC: Receiver operating characteristic
